# Feasibility of intraoperative motor evoked potential monitoring during tethered cord surgery in infants younger than 12 months

**DOI:** 10.1007/s00381-021-05316-3

**Published:** 2021-10-04

**Authors:** Johannes Herta, Erdem Yildiz, Daniela Marhofer, Thomas Czech, Andrea Reinprecht, Karl Rössler, Klaus Novak

**Affiliations:** 1 Department of Neurosurgery, Medical University of Vienna, Vienna, Austria; 2 Department of Otorhinolaryngology, Medical University of Vienna, Vienna, Austria; 3 Department of Anaesthesiology, General Intensive Care and Pain Management, Medical University of Vienna, Vienna, Austria

**Keywords:** Intraoperative monitoring, Motor evoked potential, Tethered cord, Pediatric neurosurgery

## Abstract

**Purpose:**

Feasibility, reliability, and safety assessment of transcranial motor evoked potentials (MEPs) in infants less than 12 months of age.

**Methods:**

A total of 22 patients with a mean age of 33 (range 13–49) weeks that underwent neurosurgery for tethered cord were investigated. Data from intraoperative MEPs, anesthesia protocols, and clinical records were reviewed. Anesthesia during surgery was maintained by total intravenous anesthesia (TIVA).

**Results:**

MEPs were present in all patients for the upper extremities and in 21 out of 22 infants for the lower extremities. Mean baseline stimulation intensity was 101 ± 20 mA. If MEPs were present at the end of surgery, no new motor deficit occurred. In the only case of MEP loss, preoperative paresis was present, and high baseline intensity thresholds were needed. MEP monitoring did not lead to any complications. TIVA was maintained with an average propofol infusion rate of 123.5 ± 38.2 µg/kg/min and 0.46 ± 0.17 µg/kg/min for remifentanil.

**Conclusion:**

In spinal cord release surgery, the use of intraoperative MEP monitoring is indicated regardless of the patient’s age. We could demonstrate the feasibility and safety of MEP monitoring in infants if an adequate anesthetic regimen is applied. More data is needed to verify whether an irreversible loss of robust MEPs leads to motor deficits in this young age group.

## Introduction

During spine surgery, continuous assessment of the functional integrity of motor pathways is possible by intraoperative neuromonitoring (IONM). By giving immediate feedback to the neurosurgeon, IONM reduces the risk of iatrogenic injury to the corticospinal tract and nerve roots [1]. To date, mainly two techniques are used to monitor the corticospinal tract intraoperatively: transcranial motor evoked potentials (MEPs) induced by transcranial electrical stimulation and D-wave monitoring [2, 3]. While the D-wave is recorded directly from the spinal cord via an epidural electrode after single-pulse transcranial electrical stimulation, MEPs are elicited by transcranial electrical multi-pulse stimulation and recorded from needle electrodes placed into limb muscles. Although D-wave monitoring provides the most reliable information to assess the integrity of the corticospinal tract in adults, it seems to have no application in children younger than 21 months due to the immaturity of the corticospinal tract [4]. In the case of IONM for tethered cord surgery, not only monitoring of corticospinal tract integrity but also of spinal roots is requested, since the pathology of tethering as well as the site of potential surgical injury might be located distal to the conus medullaris. MEPs have been occasionally described to be elicitable in children under the age of 12 months following transcranial electric stimulation as well as transcranial magnetic stimulation [4–10]. With younger age baseline stimulus thresholds to successfully elicit MEPs are higher than in adults [8, 11] and associated with lower reliability [12]. Recently Yi et al. published promising data that showed the feasibility of MEPs during cranial and spinal surgery in infants less than 3 months of age [10].

The objective of the present study was to make a further contribution to the topic of MEP monitoring in pediatric neurosurgery by retrospectively analyzing MEP data collected during tethered cord surgery in children younger than 12 months.

## Methods

### Patient characteristics

A retrospective chart review was conducted for all pediatric patients younger than 12 months who underwent tethered cord surgery with MEP monitoring between 2010 and 2019, at a tertiary referral hospital (Vienna General Hospital, Medical University of Vienna). Data was gathered from the medical records: demographic information and pre- and postoperative clinical evaluation (up to 3 years), operative records, and IONM protocols.

### Anesthesia protocol

Infants received in most cases intravenous premedication with midazolam. Induction of anesthesia was accomplished by an intravenous bolus of fentanyl and propofol. If no peripheral IV catheter was in place, inhaled sevoflurane was used for induction of anesthesia and immediately exchanged for propofol after IV access was given. Prior to intubation, a short-acting muscle relaxant (rocuronium) was administered. The anesthesiologist was advised that any further administration of muscle relaxants should be avoided during the operation and had to be reported to the IONM team. Anesthesia was maintained by continuous intravenous administration of propofol adapted to the weight of the infant and adjusted to the hemodynamic situation. Analgesia was achieved by continuous administration of remifentanil. To detect possible confounders in MEP monitoring, which might be caused by the anesthetic regimen, a retrospective chart review was conducted. Anesthesia protocols were screened for (1) dosage and time points of administered anesthetics and muscle relaxants, (2) arterial blood pressure levels at IONM baselines, and (3) anesthetic urgencies such as reanimation or a sudden insecure airway.

### MEP stimulation and monitoring protocol

MEPs were obtained using a NIM-Eclipse® monitoring system ver. 3.5.354 (Medtronic XOMED Inc., Memphis, TN, USA). Transcranial electrical stimulation was carried out via needle electrodes that were placed subdermally on the scalp at C3/C4 and C1/C2 according to the international 10–20 system. The montage used for stimulation of MEPs (C3/C4 or C1/C2) depended on the quality of recorded signals as well as on motion artifacts induced by transcranial stimulation. By switching the polarity of stimulation left and right, MEPs were selectively generated. A train of 5 pulses, each with a duration of 500 µs, at 250 Hz and a frequency of trains at 0.5–1 Hz was used. If necessary, the count of pulses within the train was increased to a maximum of 9 pulses. Intensity up to 200 mA was used to elicit MEPs. MEPs were recorded using needle electrodes in all 4 extremities, including at least abductor hallucis muscles and tibialis anterior muscles as well as abductor pollicis brevis muscles as a control modality. Depending on the anatomy of the pathology, additional muscles were added: quadriceps femoris, tibialis anterior, gastrocnemius, biceps brachii, extensor digitorum communis, and external anal sphincter muscles. The bandpass filter was 200–1500 Hz, and the time base was set at 100 ms. Baseline MEPs were recorded at the lowest intensity to reach motor response threshold. MEP settings were recorded at the beginning (baseline), and at the end of each operation, MEPs were monitored continuously during critical periods of the operation, if stimulation-induced patient movement did not interfere with the procedure. Events were immediately reported to the surgeon.

In addition to MEP monitoring our institutional IONM protocol for tethered cord surgery involves somatosensory evoked potential (SSEP) monitoring of the median and tibial nerve, bulbocavernosus reflex (BCR) monitoring, and lumbar and sacral nerve root mapping.

### Analysis of MEPs

If MEPs were present, the lowest stimulation intensity (mA) used to elicit a MEP response before dural opening (baseline) and after dural closure or laminoplasty was compared by semiquantitative analysis. If 50% of stimulated muscles under the neurosurgical site could be elicited, monitoring was considered feasible. MEPs were defined as robust if intensity increases of less than or equal to 50% compared to baseline values were needed to elicit a muscle response with the same amplitude. Accordingly, unsteady MEPs were defined by an intensity increase of more than 50% up to a maximal stimulation intensity of 200 mA. A decline in motor function was supposed if previously present MEPs were lost at dural closure.

To differentiate, an immature central nervous system from other factors that may confound MEP monitoring univariate and multivariate regression analysis was performed. The Pearson correlation coefficient: *r* with a 95% confidence interval (CI) and regression analysis between baseline intensities from abductor pollicis brevis muscles and possible influencing factors like sedation dose, use of muscle relaxants, mean arterial blood pressure levels at baseline, patient weight, and patient age was conducted with a significance level of 0.05. Patients with preexisting motor deficits of the abductor pollicis brevis muscle and/or lesions above the lumbosacral level were excluded from this analysis to exclude possible influences from pathologies.

## Ethics

The institutional review board approved the study protocol (Ethics Committee Medical University of Vienna, EK No.: 1402/2016). Because of the retrospective nature of the study, no informed consent by the legal guardians was needed.

## Results

### Patient characteristics

In the study, a total of 22 infants between 13 and 49 weeks (mean 33 ± 9 weeks) of age and a mean body weight of 7.92 ± 1.51 kg at the time of surgery were identified. All infants included in the study underwent MEP monitoring during tethered cord surgery. In all patients but one untethering took place at a thoracolumbar level due to spinal dysraphism. One patient was operated at the level of C3 to Th1 for tethered cord due to an arachnoid cyst. Patient characteristics including IONM results and clinical findings are illustrated in Table 1.

**Table 1:** Patient characteristics, clinical findings, and IONM results of 22 children under the age of 12 months who underwent surgery for tethered cord

**Case No.**	**Age (weeks),** **sex**	**Disease**	**MEP** **feasible**			**MEP** **robust**	**MEP** **lost**	**IONM outlook: decline in motor function expected?**	**Decline in motor function at hospital dismission**
			Upper limb		Lower limb				
1	40, M	LMC	Yes	Yes		Yes	No	No	No
2	35, M	SCM I	Yes	Yes		Yes	No	No	No
3	32, F	SCM I	Yes	Yes		No	No	Possible	No
4	34, M	LMC	Yes	Yes		Yes	No	No	No
5	20, F	LMC	Yes	No		No	No	Unknown	No: improved weakness of forefoot
6	28, M	LMC	Yes	Yes		Yes	No	No	No
7	17, F	SCM II	Yes	Yes		Yes	No	No	No
8	26, M	MMC	Yes	Yes		Yes	No	No	No: preexisting weakness lower limbs
9	35, M	LMC	Yes	Yes		Yes	No	No	No
10	25, F	MMC	Yes	Yes		No	No	Possible	No
11	38, M	Dermal sinus	Yes	Yes		Yes	No	No	No
12	44, M	SCM II	Yes	Yes		Yes	No	No	No
13	22, F	Arachnoid cyst (C3-Th1); postnatal lumbar MMC repair	Yes	Yes		No	No	Possible	No: preexisting paralysis of lower limbs
14	13, F	Dermal sinus	Yes	Yes		Yes	No	No	No
15	29, M	LMC	Yes	Yes		No	Yes	Yes	No: preexisting paresis left lower limb
16	40, M	LMC	Yes	Yes		Yes	No	No	No
17	49, M	LMC	Yes	Yes		Yes	No	No	No
18	40, F	LMC	Yes	Yes		Yes	No	No	No
19	42, F	Dermal sinus	Yes	Yes		Yes	No	No	No
20	30, F	LMC	Yes	Yes		Yes	No	No	No
21	47, M	LMC	Yes	Yes		Yes	No	No	No
22	36, M	LMC	Yes	Yes		Yes	No	No	No

*IONM* intraoperative neuromonitoring, *LMC* lipomyelocele, *MMC* myelomeningocele, *MEP* motor evoked potentials, *SCM* split cord malformation

### Anesthesia

Eight patients received intravenous premedication with midazolam (0.08 ± 0.21 mg/kg). Anesthesia was induced by a bolus of fentanyl (0.08 ± 0.17 mg/kg) and propofol (60 ± 24.36 mg). In only two cases, inhalation of sevoflurane had to be used. In all but three cases, the muscle relaxant rocuronium was administered prior to intubation with a mean dose of 0.74 ± 0.49 mg/kg. Four patients needed reversal of neuromuscular blockade by sugammadex prior to skin incision to guarantee sufficient muscle responses. During the operation and adapted to the hemodynamic situation, anesthesia was maintained by propofol with an overall mean dose of 123.5 ± 38.2 µg/kg/min. Analgesia was achieved by the continuous administration of remifentanil (mean infusion rate 0.46 ± 0.17 µg/kg/min). Under this sedative regimen mean arterial blood pressures of 57 ± 6 mmHg, averaged per patient over the entire course of the operation were measured. No anesthetic urgencies were encountered.

### SSEP, BCR, and nerve root mapping

SSEP monitoring of the median and tibial nerve was feasible in 19/22 (86.36%) and 17/22 (77.27%) patients. Monitoring of BCR was available in 21 patients and feasible in 13 (61.90%) patients. Lumbar and sacral nerve root mapping was successfully used in 18 (94.74%) patients and was not feasible in only one patient.

### MEP analysis

**Fig. 1:**
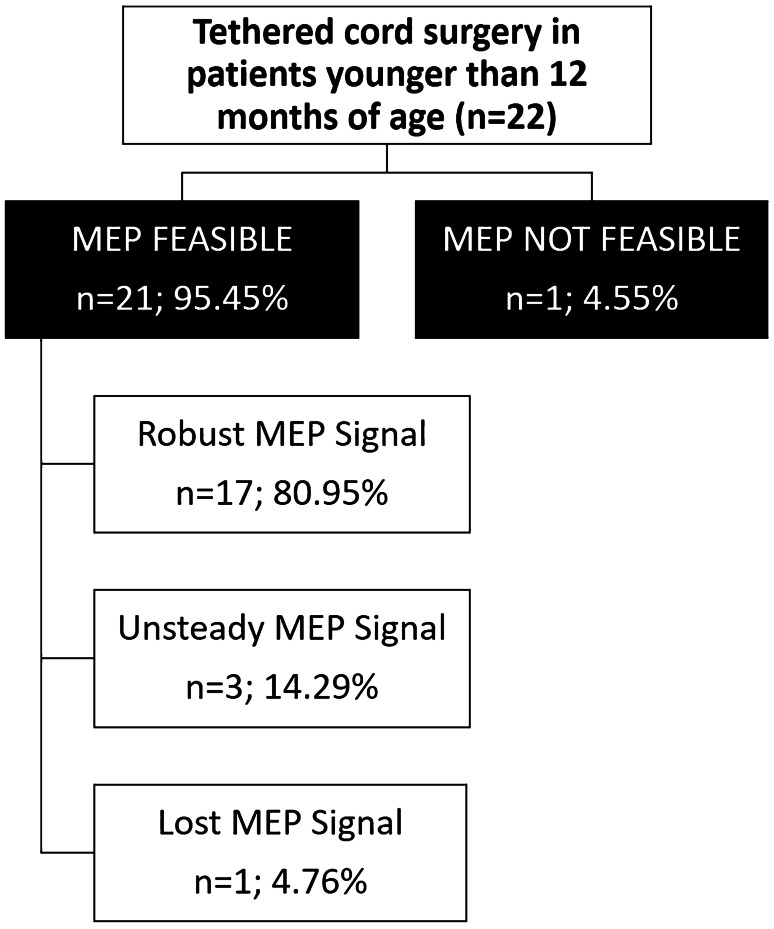
Intraoperative Motor evoked potential (MEP) changes and postoperative motor status

In all patients, robust MEP signals could be elicited in the upper extremities during the entire course of surgery giving a success rate of 100% as shown in Table 1. In the lower extremities, MEPs were present in 21 out of 22 patients giving a success rate of 95.45%. Out of these 21 patients, 17 (80.95%) patients showed a robust signal until the end of surgery (Fig. 1).

In total, MEP monitoring was considered as feasible in 21 (95.45%) patients. One patient with a preexisting weakness of both forefeet and lower legs (Case 5) did not reveal any MEPs from the lower extremities. Since robust MEPs in the upper extremities were present, the preexisting motor deficit could be causative for the missing MEPs.

**Fig. 2:**
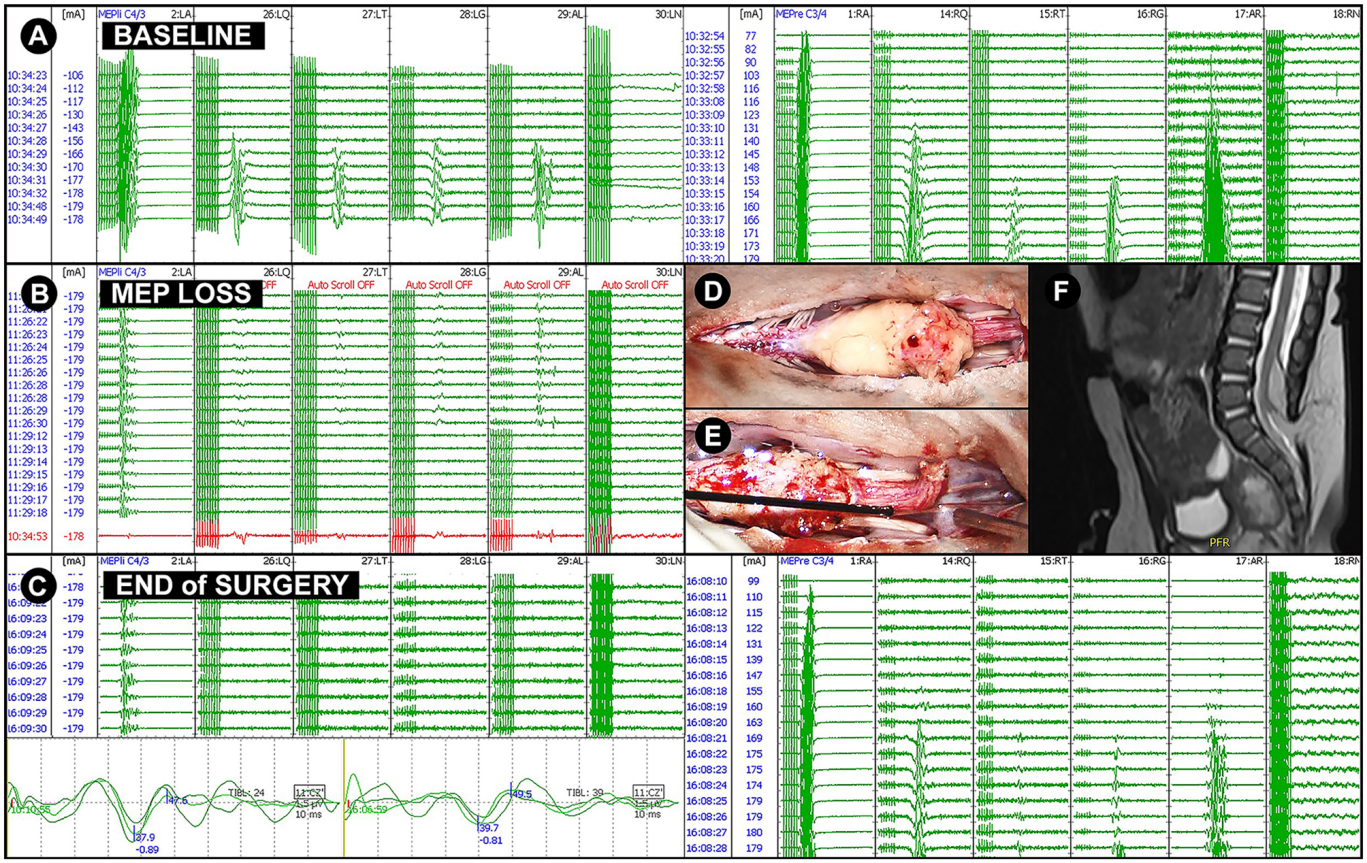
CASE 15: 29-week-old male with a slight paresis of the left lower limb. Spinal dysraphism was diagnosed already intrauterine by organ screening and fetal MRI. Preoperative MRI revealed a tethered cord due to a lipomyelocele and non-fusion of S2 (**F**). During surgery, high stimulation intensities were needed to elicit MEPs of the left lower extremities (≈160 mA) at baseline (**A**). During surgery, left-sided MEPs were instable and were irreversibly lost (**B**, **C**) after lumbar and sacral nerve roots were detached from the lipoma (**D**, **E**). There was no decline in postoperative muscle function till the last follow-up three years after surgery. AL/AR, left/right abductor hallucis; LA/RA, left/right abductor pollicis brevis; LG/RG, left/right gastrocnemius; LN/RN, left/right external anal sphincter; LQ/RQ, left/right quadriceps femoris; LT/RT, left/right tibialis anterior; MEP, transcranial motor evoked potentials

If MEPs were present till the end of surgery, no new postoperative muscle weakness occurred regardless of whether MEPs were robust or unsteady. In the one patient (Case 15) where MEPs were lost during surgery, a slight weakness of the left lower limb was already present preoperatively. High intensities were needed at the baseline to elicit MEPs (Fig. 2). The preexisting weakness, however, did not decline after surgery.

In three patients, at least one MEP under the neurosurgical site was rated as unsteady. In all three cases, this was an isolated finding of only one muscle group. All other muscle groups under the neurosurgical site remained robust during monitoring. None of these patients showed postoperative clinical deterioration in motor function.

Success rates of MEPs specified by each stimulated muscle and corresponding baseline intensities are shown in Table 2. The average intensity needed to elicit a MEP response at baseline was 101 ± 20 mA (71 ± 13 V).

**Table 2:** Success rate for baseline MEPs and minimal required stimulation intensities for corresponding muscles (*low number of applications)

**Success rate (%)**	Baseline intensity (mA)	Baseline voltage (V)	Stimulated muscle	Number of applications (*n*_max_ = 44)
**98**	78 ± 20	58 ± 12	Abductor pollicis brevis	40
**93**	124 ± 26	89 ± 19	Tibialis anterior	44
**90**	122 ± 25	88 ± 19	Gastrocnemius	42
**84**	119 ± 27	87 ± 19	Abductor hallucis	44
**83**	114 ± 22	84 ± 18	Quadriceps femoris	41
100*	71 ± 12	46 ± 6	Extensor digitorum	2
100*	76 ± 6	49 ± 3	Biceps brachii	2

**Fig. 3:**
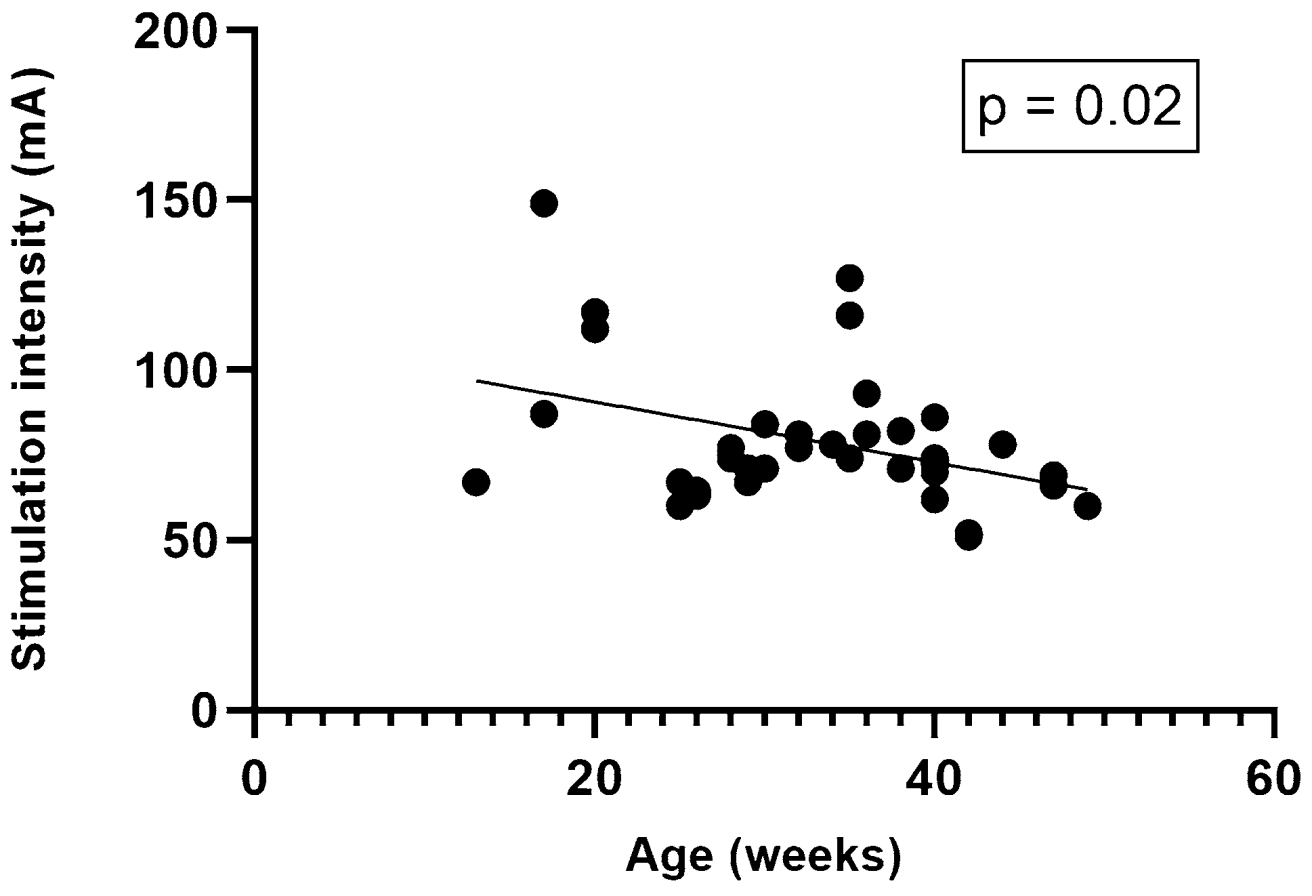
Correlation between MEP stimulation intensities of abductor pollicis brevis muscles at baseline with patients age

Linear regression analysis between the minimal stimulation intensity needed to record a reproducible MEP signal from abductor pollicis brevis muscles and (1) the administered propofol dose (*r* = 0.099, 95% CI −0.232 to 0.410, *p* = 0.558), (2) administered rocuronium dose before the start of surgery (*r* = −0.184, 95% CI −0.479 to 0.149, *p* = 0.251), (3) mean arterial blood pressure values (*r* = 0.271, 95% CI −0.069 to 0.554, *p* = 0.116), (4) patient weight (*r* = −0.141, 95% CI −0.445 to 0.192, *p* = 0.405), and (5) administered remifentanil dose (*r* = −0.028, 95% CI −0.349 to 0.299, *p* = 0.872) revealed no significant correlation. However, patient age revealed a significant association (*r* = −0.381, 95% CI −0.628 to −0.065, *p* = 0.020*) in the univariate analysis (Fig. 3) that could not be reproduced in multivariate analysis (95% CI −2.441 to 0.066, *p* = 0.063).

No complications were caused by intraoperative monitoring of MEPs.

## Discussion

### Feasibility and thresholds of MEPs

Neurosurgical procedures in tethered cord syndrome convey the risk of adverse neurologic sequelae like motor weakness and bladder dysfunction. To prevent surgically induced disabling motor injury, MEP monitoring is used. Lately, this technique has been applied more frequently also in pediatric patients, but reports in the age group under 12 months are still scarce and mostly refer to older children [4, 8, 10, 11, 13–17].

In our study, we could show that MEP monitoring was feasible in all patients for the upper extremity and in 21 out of 22 patients for the lower extremities. The one patient in whom MEPs could not be elicited in the lower extremities suffered from preexisting paresis of the lower limbs. This finding is comparable with the good results of a recently published work by Yi et al [10]. The authors reported successful MEP monitoring in 24 out of 25 children under 3 months of age that underwent neurosurgical procedures for mixed etiologies. Other authors have published similar promising results in this young age group [8, 17]. Fulkerson et al. could show reliable MEP recordings from all 4 extremities in only two of three children under 12 months (in a cohort of 10 children between the age of 5 to 31 months) [8]. They found stimulation thresholds to be higher than in adults with baseline threshold voltages greater than 400 V. This negative correlation between age and baseline MEP stimulation thresholds has already been described by Lieberman in 2006 [11]. They studied the effect of age on MEPs in 56 children aged 2 to 18 years who underwent spine surgery under propofol but also isoflurane anesthesia. Feasible MEPs were defined by a MEP amplitude of 50 µV or greater in all monitored muscle groups. Minimum threshold voltage was analyzed prior to surgical incision. Younger age was associated with increased threshold voltage, longer stimulating pulse trains, and the greater need to adjust stimulating scalp electrodes.

The prevailing explanation of the clinical observation that higher stimulation intensities are needed in children to elicit a MEP response is given by an immature corticospinal tract that leads to low conduction velocities [18]. Myelination initiates in the second trimester of pregnancy and lasts beyond puberty [19, 20] In several studies using transcranial magnetic stimulation, it could be shown that minimum required stimulation intensities to elicit MEPs did not decrease until the 2nd decade of life [6, 21, 22].

Sala et al. reviewed MEP data of 112 children who underwent neurosurgery [15]. In 94 children, mean threshold intensities to elicit upper extremity MEPs were 74 ± 34.3 mA (range of 30 to 180 mA). Threshold comparisons revealed that younger children required higher threshold intensities. This was not statistically significant when thresholds in children under the age of three years were compared with older children. In our noticeable young age group, a significant correlation between younger age and higher threshold intensities to elicit MEPs was found in univariate analysis but not in multivariate analysis. With an overall mean threshold intensity of 101 ± 20 mA and a mean threshold intensity of 78 ± 20 for the abductor pollicis brevis muscle, our data are consistent with the previously mentioned study by Sala et al. [15]. This data suggests that threshold intensities in the young are higher but do not differ significantly from adults. Furthermore, stimulating the motor cortex in infants is facilitated by a lower impedance due to a thinner skull. We, therefore, hypothesize that high stimulation thresholds described by others may be due to different stimulation parameters such as pulse duration [8, 10]. Increasing the amount of stimuli in the trains as well as pulse duration leads to better temporal summation at the lower motor neuron and therefore may overcome the lack of synchrony which is given in an immature corticospinal tract [4]. The same is true for techniques like double train facilitation or spatial facilitation techniques that have not been used in our study [10, 14, 17, 23].

Based on the theory of cortical representation and in accordance with the findings of Khater-Boidin et al., that, even in newborns, MEPs of the upper extremities can be elicited; baseline intensity thresholds for the thumb muscles were the lowest and showed the highest success rates in our cohort [5]. As described by Sala et al., it is advised to select distal muscles like the small hand muscles, long forearm flexors or extensors, and small and long flexors of the foot as they have a stronger innervation than proximal muscles [15, 24].

## Prognostic value of MEPs

During surgery, the surgeon was informed about threshold elevations and loss of MEPs. If MEP thresholds were unsteady (intensity increase >50%), we assumed temporary paresis of the associated muscle. This arbitrary definition was used as a warning criterion to the surgeon and is based on our experience that the best-innervated muscle, namely, the abductor pollicis brevis muscle, usually needed stimulation intensities between 60 and 110 mA in children. An intensity increase of 50%, therefore represents a situation in which sufficient monitoring is still possible in the majority of cases. All three cases where unsteady MEPs occurred were isolated findings of only one muscle group. Out of the three patients, two did not reveal any postoperative weakness while the third patient already suffered from a preoperative weakness in the affected muscle.

If MEPs disappear irreversibly, we assume a new postoperative weakness that can be temporary or permanent as suggested in the literature [1, 25, 26]. MEP loss was observed in one case (Case 15; Fig. 2). The patient already suffered from a preexisting weakness in the left lower limb that did not worsen after surgery. Here, it is important to note that (1) stimulation thresholds were already high at baseline with a train of 9 stimuli, (2) “anesthetic fade” might have occurred and obscured the results as higher thresholds of the abductor pollicis brevis muscle could be seen at the end of surgery [27], and (3) precise clinical assessment and muscle strength grading are impossible in a 29-week-old child and further obscured by the retrospective nature of the study. On the other hand, Case 5 who preoperatively suffered from a weakness of the lower extremities did not reveal MEPs in the lower extremities while presenting with robust MEPs from the upper extremities. This in turn might indicate that, even in young children, MEP monitoring is sensitive to injury of the corticospinal tract as described in adults [13]. Due to the low number of MEP changes in our study conclusions about the prognostic relevance of MEPs are limited. Yi et al. did not encounter any false-negative results in 19 patients in whom intensities remained under 50% of the baseline amplitude [10]. From the four patients that had an irreversible increase of 50% and greater, one was lost during follow-up; one had no deficit and two showed postoperative weakness. Fulkerson et al. defined a complete loss of signal or a decrease in MEP amplitude greater than 50% as a warning criterion for a postoperative neurological deficit [8]. Two patients had a persistent decline of MEPs, and the postoperative clinical status matched the prognosis of postoperative motor deficit. Due to the small amount of data available in the examined age group and the large variability of used stimulation parameters and definitions of warning criteria, it is currently not possible to make a precise statement about the reliability of MEPs in young children.

## Anesthesia

It has been well described that various anesthetic drugs may influence MEP recordings. Especially inhalation agents like sevoflurane cause more suppression of lower motor neuron excitability in comparison with total intravenous anesthesia [28–33]. Therefore, the use of stable intravenous anesthesia with propofol and opioids (TIVA) has been recommended during MEP monitoring [24] and has been successfully applied in children [8, 10, 12, 17, 34]. In our study in only two cases, sevoflurane was used to initiate anesthesia. After intravenous lines were in place, sevoflurane was immediately switched to propofol and remifentanil. This approach did not show any negative effect on MEP thresholds. Because deepening of anesthesia may reduce or even extinguish MEP recordings, the anesthesiologist was advised at the beginning of surgery not to administer bolus doses of propofol. In young children, titrated doses of 100–250 µg/kg/min of propofol have been described to show good MEP success rates, which has been confirmed by our study (mean propofol dosage 123.5 ± 38.2 µg/kg/min) [8]. It has been shown that younger children require higher doses of propofol to achieve adequate anesthesia [11, 35, 36]. One could hypothesize that larger doses of propofol lead to higher MEP threshold intensities and thus contribute to insufficient MEP monitoring. Neither could we show any correlation between the needed stimulation intensity to record a reproducible MEP signal and the dosage of continuous infused propofol or remifentanil. Nor could we show any significant correlation between age and the amount of administered propofol. An explanation for this could be given by the fact that only a limited number of patients with a slight age difference was investigated.

Other frequently used drugs that prevent MEP monitoring are drugs that cause neuromuscular blockade [29]. In our study, neuromuscular blockade was performed during intubation in the majority of patients but was not repeated afterward. Drug effects were therefore extinguished in all, but four patients when MEP baselines were established. In the four remaining cases, sugammadex, a drug that leads to reversal of neuromuscular blockade, was used to in order to allow MEP monitoring.

## Safety

If not used conscientiously, transcranial electric stimulation can also be a source of danger. It harbors several risks like burns and bleeding at electrode sides, bite injury to the tongue, lip and ventilation tube, arrhythmia, triggering of seizures as well as body movement induced injury during delicate surgical steps [37]. None of the mentioned complications was encountered in our patient cohort. We therefore assume that intraoperative MEP monitoring is sufficiently safe in infants younger than 12 months if carried out by an expert group. To minimize the risks of our young patients we take the following precautions: (1) monitoring is carried out by a biomedical analyst with several years of IONM experience and an experienced neurophysiologist; (2) during IONM well-coordinated communication with the surgeon is pivotal. The surgeon is warned prior to MEP monitoring if excessive movement is encountered at baseline, (3) to prevent bite injuries a bite block is routinely used, and (4) for transcranial stimulation in patients with open skull sutures, we used subdermal needle electrodes instead of corkscrew electrodes.

## Limitations

The study has all inherent limitations associated with retrospective data collection in a relatively small patient cohort. While IONM data and anesthesia data were extracted from well-documented databases, collection and evaluation of pre- and postoperative motor status were difficult to ascertain in this young age group. Furthermore, comparisons of our results with other studies are rather not useful due to different MEP stimulation techniques, anesthesia protocols, and high trial-to-trial variability. Here, a consensus finding and standardization in the technology to be used would be necessary.

## Conclusions

MEP monitoring has shown to be safe and feasible in children under the age of 12 months during untethering procedures with the described stimulation parameters and anesthetic regimen. Furthermore, our data suggest that MEP monitoring should be used routinely in young children even in the presence of preoperative motor deficits. If MEPs are present till the end of surgery, no new postoperative motor deficits are expected. More data is necessary to ensure prognostic outcome assessment, especially in cases of irreversible MEP loss.

## Abbreviations

BCR: Bulbocavernosus reflex
CI: Confidence interval
IONM: Intraoperative neuromonitoring
MEP: Motor evoked potential
SSEP: Somatosensory evoked potential
TIVA: Total intravenous anesthesia
